# Unsupervised industrial image defect detection based on autoencoder and GANs

**DOI:** 10.1371/journal.pone.0346637

**Published:** 2026-04-10

**Authors:** Shuangli An, Junjie Wu, Jiawang Li

**Affiliations:** School of Intelligent Manufacturing and Control Engineering, Shanghai Polytechnic University, Shanghai, China; Northwestern Polytechnical University, CHINA

## Abstract

In the process of intelligentization in modern manufacturing, especially in industrial fields such as automobile manufacturing, semiconductor production, and electronic product assembly, product quality control is crucial. Traditional defect detection methods face problems such as supervised learning methods relying on a large amount of labeled data, weak generalization ability, and high cost. In the process of intelligent manufacturing, industrial product quality control is a key link to ensure production safety and product consistency. Especially in typical industrial scenarios such as automobile manufacturing, semiconductor production, and electronic product assembly, traditional defect detection methods are difficult to meet the needs of actual production lines for high-precision and high-efficiency detection due to their reliance on a large amount of labeled data, weak generalization ability, and high cost. To solve the problem of defect detection in small and zero sample scenarios in these industries, improve detection sensitivity and localization accuracy, and enhance the model’s generalization ability to unknown defects, a unsupervised industrial image defect detection method based on autoencoder and Generative Adversarial Networks (GANs) is proposed. This study constructs an Multi-level Deep feature Adaptive fusion AutoEncoder (MDAAE) module, extracts multi-scale features through Pre-Trained Convolutional Neural Backbone Networks (PTCNBN), introduces Attention Mechanism (AM) to dynamically calculate feature weights, and achieves feature fusion and reconstruction. Meanwhile, the self-AM is fused to improve the GANs. The self-attention module efficiently captures long-range dependencies and generates an adversarial network training objective function to optimize the generator’s unsaturated loss. The results showed that the Area Under the Curve (AUC) of the Recall-Precision curve of the research method reached 93.6 ± 0.5%, and its F1-Score value exceeded 0.890 ± 0.003 in defect types such as scratches and dents. In practical applications, the inference time of the research method remained stable at 3.0 ± 0.2 GB of central processor memory, and the Fréchet Inception Distance (FID) value fully converged at the 2230th iteration, with a stable FID value of 3. The false alarm rate was only 8.6 ± 0.7% under strong light conditions. The proposed UIIDD method based on autoencoder and GANs had good robustness, generalization ability, reliability, and efficiency. This study effectively solves the problems of poor robustness, weak generalization ability, and high cost of traditional defect detection methods.

## 1. Introduction

In the growth of modern manufacturing towards intelligence and automation, product quality control plays a crucial role [1]. For example, in scenarios such as automobile body welding, semiconductor wafer inspection, and textile surface quality inspection, the diversity and randomness of defects make traditional methods difficult to cope with. In specific tasks such as automotive welding seam detection, semiconductor wafer micro defect recognition, and textile defect localization, the defect morphology is diverse, the frequency of occurrence is low, and it is often accompanied by complex environmental interference such as lighting changes and material differences, making it difficult for traditional visual systems to work stably. However, traditional defect detection methods often face the challenge of obtaining a large number of qualified products in practical applications. Defect samples with diversity and randomness are often scarce, difficult to collect, and even some types of defects are completely unknown during the training phase [2]. The defect detection scenarios with small or zero samples make it difficult for supervised learning methods to hold strong assumptions that require many labeled defect data for training, resulting in weak model generalization ability and poor detection ability for unknown defect types. In addition, the high cost of manual annotation seriously restricts the deployment efficiency and applicability of detection systems [3]. Unsupervised learning methods have become an attractive research direction in Industrial Defect Detection (IDD) due to their characteristic of not relying on defect sample labels. Among numerous unsupervised methods, autoencoders have powerful feature learning and data reconstruction capabilities, which can compress the input image into a low-dimensional latent space through learning and reconstruct it back to the original image [4]. GANs have excellent image generation capabilities, which can learn and approximate complex high-dimensional data distributions through adversarial training of generators and discriminators [5]. Therefore, to improve the sensitivity, localization precision, generalization, and robustness of UIIDD systems, this paper innovatively proposes a UIIDD method built on autoencoders and GANs. During the research process, a MDAAE is constructed. It extracts Multi-level Deep Feature (MLDF) through a PTCNBN, calculates feature weights using Global Average Pooling (GAP) and AM, and compares the local structural similarity between the two pixel by pixel. A self-AM is introduced to improve GANs and learn the normal sample distribution through an encoder-decoder structure with a self-attention module. Using a self-AM-enhanced encoder, the authenticity of the generated image is judged by combining latent vectors, and the deviation from the normal mode is quantified by calculating pixel-level anomaly scores. The combination of the two ultimately achieves precise defect localization. It is expected that the designed method can offer theoretical support for improving the efficiency and generalization of UIIDD measurement.

## 2. Related works

In industrial production, there are numerous types and shapes of defect samples, with extremely low frequency and unpredictability. Collecting and annotating a lot of samples covering all possible types and shapes of defects is extremely costly. Therefore, research on UIIDD is meaningful. Tao X et al. designed a joint feature reconstruction and repair method built on a Siamese framework to address the problem of poor performance in Unsupervised Anomaly Detection (UAD) in industrial scenarios due to unpredictable defects and limited discriminative information. This study captured discriminative features of normal and generated defective samples through Siamese networks, which could effectively model the distribution of normal features [6]. Wan Q et al. proposed an unsupervised image anomaly detection and segmentation framework based on pre-trained feature mapping to handle the scarcity of abnormal data in automatic product quality detection in intelligent manufacturing. The research process was validated on the MVTec AD dataset. This method outperformed existing techniques in both detection accuracy and computational efficiency [7]. Tang B et al. developed an automatic detection method built on machine vision for surface defects in steel products. The research method had the advantages of high efficiency, high degree of automation, and strong adaptability [8]. Maggipinto et al. proposed a UAD method based on a convolutional autoencoder to address traditional univariate control charts being difficult to capture complex multivariate anomalies in the manufacturing industry. In the testing of spectral data in semiconductor manufacturing, the research method could effectively improve the performance of anomaly detection and overcome the limitations of insufficient industrial data labeling and complex structure [9]. Cao Y et al. put forward a biased student framework based on biased knowledge generation, transfer, and fusion to address the issues of overfitting in supervised methods and neglect of abnormal bias knowledge in unsupervised methods in industrial testing. This method could effectively combine the advantages of supervised and unsupervised methods, and could effectively improve the accuracy of anomaly localization [10].

In addition, many industry scholars have conducted in-depth research and applications on autoencoders and GANs. Bao J et al. proposed a method of coupling data assimilation and depth generation models to address the geological structure identification challenges caused by strong heterogeneity and data scarcity in groundwater modeling. It compared the performance of two networks in parameterized conductivity fields through synthetic case testing. The research method could generate realistic geological structures with better positioning accuracy [11]. Khan W et al. put forth a joint model built on a dual variational autoencoder and GANs to address the lack of labeled data in anomaly detection in attribute networks. This method could effectively perform nonlinear modeling and distribution alignment [12]. Abirami S et al. proposed a generative modeling method based on autoencoders and GANs to address the issues of nonlinear spatiotemporal correlations and incomplete data in PM2.5 forecasting. This method captured data distribution through reverse learning and had strong generalization ability and high accuracy [13]. Liu et al. proposed an unsupervised detection method for autoencoder Wasserstein GANs built on continuous wavelet transform and ensemble adversarial training to address the difficulty in obtaining bearing fault samples. This method had good detection accuracy, real-time performance, and anomaly localization capability [14]. Zou C et al. established a new architecture based on deep learning that integrates autoencoders and GANs to address channel fading and one-bit quantization nonlinearity in underwater wireless optical communication. This process combined a dedicated loss function and training strategy to optimize performance, effectively addressing complex underwater environments with both high efficiency and low complexity advantages [15].

In summary, existing research has made significant progress in UIIDD, but in actual industrial scenarios (such as metal surface scratch detection, textile defect localization, etc.), it still faces problems such as poor adaptability to complex environments and limited generalization ability in small samples. Autoencoders can effectively improve their robustness to noise and interference by introducing noise or regularization terms, and they do not require labeled data, allowing them to learn useful feature representations from unlabeled data. GANs do not require complex inference processes when generating data, and can learn the true distribution of data through adversarial training of the generator and discriminator, thereby generating novel data that conforms to the true distribution of data. The paper designs a UIIDD method built on autoencoder and GANs, aiming to meet the requirements of UIIDD, lift the efficiency, robustness, and generalization capability of UIIDD technology, and enhance the economic benefits and social value of the industry.

## 3. Design of UIIDD method

### 3.1 UIIDD method based on MDAAE

**Fig 1:**
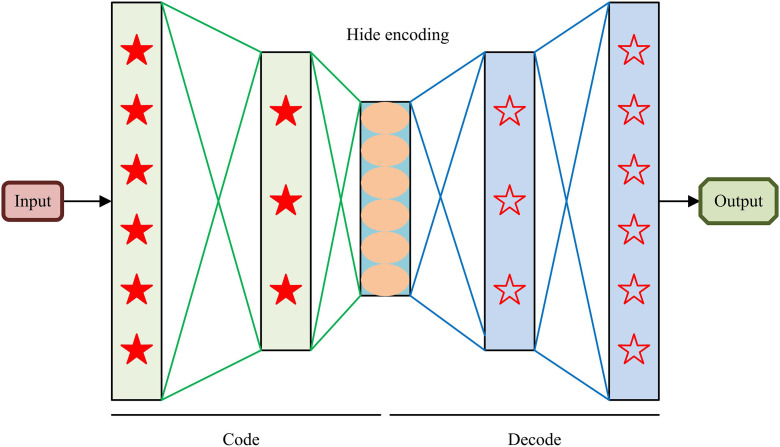
Structure of autoencoder.

The traditional UIIDD method requires manual design of feature extraction rules. However, these rules are only effective for specific types of defects, making it difficult to adapt to complex and changing industrial scenarios, and their computational efficiency is low, making it difficult to meet real-time requirements [16–17]. Autoencoders transform defect detection into an out-of-distribution detection problem, constructing a “memory bank” of normal samples through unsupervised learning, and achieving precise extraction and fusion of defect sensitive features through MLDF adaptive fusion mechanism [18–19]. Therefore, this study introduces an autoencoder for UIIDD and improves it through the MLDF adaptive fusion mechanism. Fig 1 shows the specific structure of the autoencoder.

In Fig 1, the autoencoder compresses the raw data through the encoder, generates key, low dimensional “hidden encoding”, and extracts the essential features of the data. Subsequently, the decoder uses this encoding for data reconstruction and outputs the reconstruction result. Among them, the objective function of the autoencoder includes reconstruction loss and L2 weight regularization term, as shown in equation (1).


$$J\left(\vartheta \right)=\frac{\text{1}}{n}\sum_{i=1}^{n}∥{𝐱}^{\left(i\right)}-g_{W_{g},{𝐛}_{g}}\left(f_{W_{f},{𝐛}_{f}}\left({𝐱}^{\left(i\right)}\right)\right){∥}^{\text{2}}+\lambda \cdot \frac{\text{1}}{\text{2}}\sum_{T\in \left\{f,g\right\}}∥W_{T}\overset{\text{2}}{\underset{F}{∥}}$$ (1)

In equation (1), J(ϑ) is the objective function, ϑ is the set of all learnable parameters of the model, and n is the amount of training samples. ${𝐱}^{\left(i\right)}$ denotes the i -th input sample vector, $f_{W_{f},{𝐛}_{f}}$ is the encoder function, and $g_{W_{g},{𝐛}_{g}}$ is the decoder function. $W_{f}$ / $W_{g}$ and ${𝐛}_{f}$ / ${𝐛}_{g}$ are the weight matrices and bias vectors of the encoder and decoder. λ is the regularized word count, $\overset{\text{2}}{\underset{F}{\|\cdot \|}}$ represents the square of the L2 norm, F represents the Frobenius norm of the matrix, and T is the set of layer types. Next, this study extracts multi-scale features of industrial images through PTCNBNs, and the MLDF extraction formula is shown in equation (2).


$$\{F_{l}=\sigma_{l}\left(\overset{conv}{\underset{l}{W}}*F_{l\text{-}\text{1}}\text{+}\overset{conv}{\underset{l}{b}}\right)\overset{L}{\underset{l=1}{\}}}$$ (2)

In equation (2), $F_{l}$ is the l -th layer feature map, $\sigma_{l}\left(\cdot \right)$ is the hierarchical correlation activation function, $\overset{\text{conv}}{\underset{l}{W}}$ and $\overset{\text{conv}}{\underset{l}{b}}$ are the convolution kernel weights and biases. * is the convolution operation, and L is the gross of layers. To address the limitations of manually assigning weights, this study introduces AM dynamic calculation of hierarchical weights, as shown in equation (3).


$$\alpha_{l}=\frac{exp\left({𝐯}^{T}\cdot \text{GAP}\left(F_{L}\right)/\tau \right)}{\overset{L}{\underset{k=1}{\Sigma }}exp\left({𝐯}^{T}\cdot \text{GAP}\left(F_{k}\right)/\tau \right)}$$ (3)

In equation (3), $\alpha_{l}$ is the l -layer feature fusion weight, GAP(·) is GAP, and ${𝐯}^{T}$ is the transpose of the learnable weight vector. τ is the temperature coefficient utilized to control the sharpness of the weight distribution. Finally, this study upsamples features of different scales to a unified resolution and weights them for aggregation to avoid conflicts between levels [20–21]. The expression for multi-scale feature fusion is shown in equation (4).


$$\left\{ \begin{array}{c} @l@F_{\text{fuse}}=\sum_{l=1}^{L}\alpha_{l}\cdot U_{l}\left(F_{l}\right)\\ U_{l}\left(F_{l}\right)=\text{Deconv}(F_{l};\overset{\text{up}}{\underset{l}{W}})⊕{\text{Conv}}_{1\times 1}(F_{l};\overset{\text{proj}}{\underset{l}{W}}) \end{array}\right.$$ (4)

**Fig 2:**
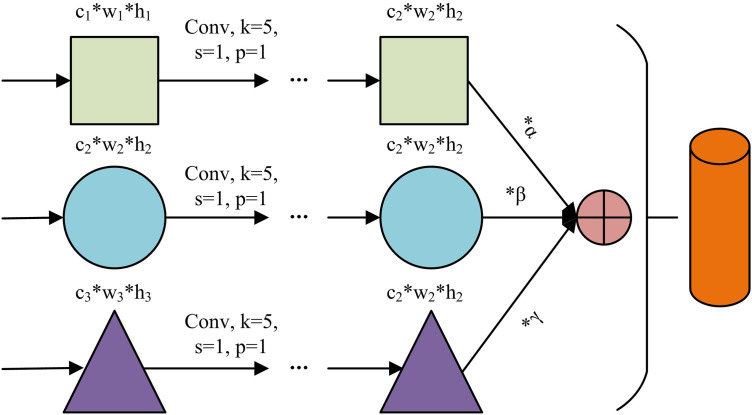
MLFF module diagram.

In equation (4), $F_{\text{fuse}}$ is the fusion feature and $U_{l}(\cdot )$ is the feature unification operation. $\overset{\text{up}}{\underset{l}{W}}$ and $\overset{\text{proj}}{\underset{l}{W}}$ are upsampling kernels and projection convolution kernels, ⊕ is feature concatenation operation, and Deconv(·) is deconvolution operation. ${\text{Conv}}_{1\times 1}(\cdot )$ is the 1×1 convolution operation. In summary, the Multi-Level Feature Fusion (MLFF) module diagram of industrial images is shown in Fig 2.

In Fig 2, the module processes input features at different levels through three parallel paths. After aligning the dimensions of each path through a convolutional layer with unified parameters, a differential weighting strategy is used for fusion, and the fused features are merged into the output. After the multi-level features of industrial images are fused, this study further compresses the fused features. The encoder compresses and fuses the features into the hidden space bottleneck layer, and the decoder reconstructs the features, forcing the network to learn a general representation of normal features through bottleneck design [22–23]. The fusion feature reconstruction formula is shown in equation (5).


$$\left\{ \begin{array}{c} @l@{\hat{F}}_{\text{fuse}}=g_{\partial_{d}}\left(f_{\partial_{e}}\left(F_{\text{fuse}}\right)\right)\\ f_{\partial_{e}}\left(𝐗\right)=\text{Encoder}\left(X;\{\overset{i}{\underset{e}{W}},\overset{i}{\underset{e}{b}}\overset{N}{\underset{i=1}{\}}}\right)\\ g_{\partial_{d}}\left(𝐙\right)=\text{Decoder}\left(Z;\{\overset{j}{\underset{d}{W}},\overset{j}{\underset{d}{b}}\overset{M}{\underset{j=1}{\}}}\right) \end{array}\right.$$ (5)

In equation (5), ${\hat{𝐅}}_{\text{fuse}}$ is the reconstructed fused feature, $f_{\partial_{e}}(\cdot )=\text{Encoder(}\cdot \text{)}$ and $g_{\partial_{d}}(\cdot )=\text{Decoder}\left(\cdot \right)$ are the encoding and decoding functions, 𝐗 is the fused feature input, 𝐙 is the bottleneck layer feature, and N and M are the layers of the encoder and decoder. Next, to make the hidden space more compact and enhance the sensitivity of the system to industrial image defect features, this study introduces a multi-level reconstruction loss function, whose function formula is shown in equation (6).


$$\left\{ \begin{array}{c} @l@{\text{L}}_{\text{recon}}=\sum_{l=1}^{L}\xi_{l}\overset{\text{2}}{\underset{F}{\|M_{l}⊙\left(F_{l}-D_{l}\left({\hat{F}}_{\text{fuse}}\right)\right)\|}}+\beta \cdot R_{\text{sparse}}\left(z\right)\\ {\text{D}}_{l}\left(X\right)={\text{Conv}}_{3\times 3}\left({\text{Downsample}}_{l}\left(X\right)\right) \end{array}\right.$$ (6)

In equation (6), ${\text{L}}_{\text{recon}}$ is the multi-level reconstruction loss, $\xi_{l}$ is the hierarchical loss weight, and ${\text{D}}_{l}\left(\cdot \right)$ is the feature decoding mapping. $M_{l}$ is the hierarchical effective area mask, ⊙ is element wise multiplication, and $R_{\text{sparse}}\left(z\right)$ is sparse regularization of bottleneck features. β is the sparsity regularization coefficient, and ${\text{Downsample}}_{l}\left(\cdot \right)$ is the level dependent downsampling. Finally, by capturing texture and structural anomalies, defects in industrial images are located and unsupervised defect detection is achieved. The formula for defect localization is given by equation (7).


$$\left\{ \begin{array}{c} @l@S\left(x,y\right)=\sum_{l=1}^{L}\gamma_{l}\cdot \overset{\left(x,y\right)}{\underset{l}{\text{SSIM}}}\left(F_{l},{\text{D}}_{l}\left({\hat{F}}_{\text{fuse}}\right)\right)\\ \overset{\left(x,y\right)}{\underset{l}{\text{SSIM}}}=\frac{\left(2\mu_{f}\mu_{\hat{f}}+c_{\text{1}}\right)\left(2\sigma_{f\hat{f}}+c_{\text{2}}\right)}{\left(\overset{\text{2}}{\underset{f}{\mu }}+\overset{\text{2}}{\underset{\hat{f}}{\mu }}+c_{\text{1}}\right)\left(\overset{\text{2}}{\underset{f}{\sigma }}+\overset{\text{2}}{\underset{\hat{f}}{\sigma }}+c_{\text{2}}\right)} \end{array}\right.$$ (7)

**Fig 3:**
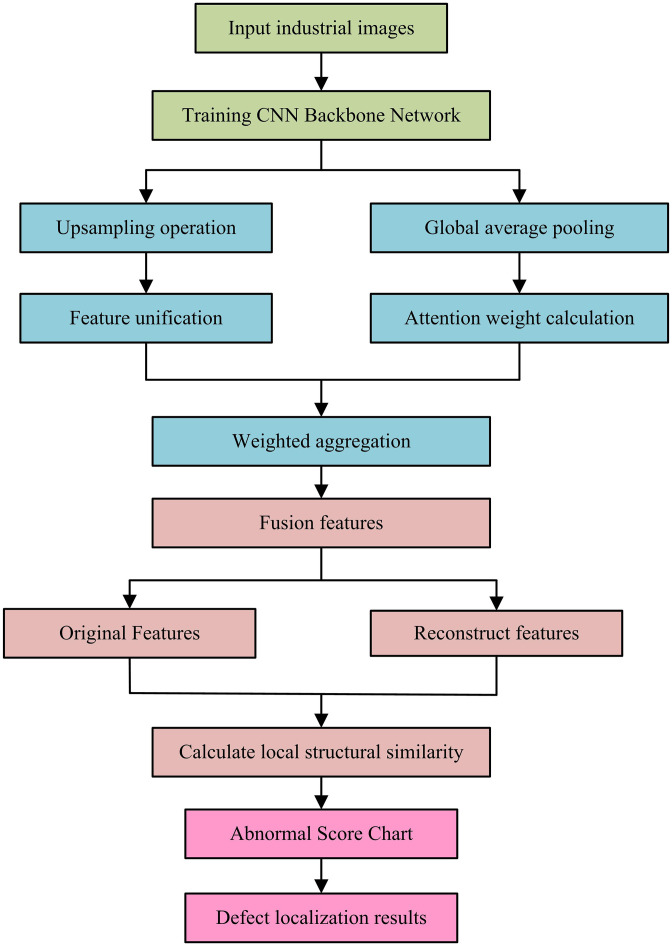
UIIDD process based on MDAAE.

In equation (7), S(x,y) means the anomaly score of pixel position (x,y), $\gamma_{l}$ is the hierarchical localization weight, and $\overset{\left(x,y\right)}{\underset{l}{\text{SSIM}}}$ is the local structural similarity. $\mu_{f}$ and $\overset{\text{2}}{\underset{f}{\sigma }}$ are the mean and variance of the local window. $\sigma_{f\hat{f}}$ is the covariance between the original and reconstructed features. $c_{\text{1}}$ and $c_{\text{2}}$ are stability coefficients. In summary, the flow of the UIIDD method based on MDAAE is shown in Fig 3.

In Fig 3, the UIIDD based on MDAAE first inputs industrial images and extracts MLDF through a PTCNBN. The next step is to split the processing into two paths: one path uses upsampling to unify the feature scale, and the other path uses GAP and AM to calculate the feature weights. The two generate fused features through adaptive weighted aggregation, which are then decoupled into original features and decoder-reconstructed features. Finally, the local structural similarities between the two are compared pixel by pixel, generating an anomaly score map and outputting the defect localization results.

### 3.2 UIIDD method integrating AM and GANs

**Fig 4:**
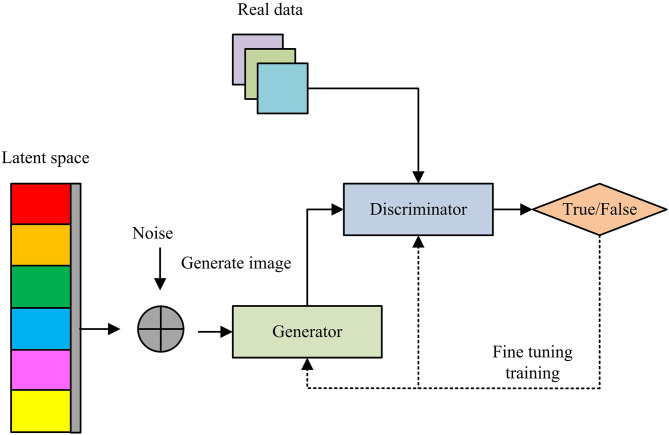
GANs training process.

The UIIDD method based on MDAAE can improve defect generalization ability, enhance small defect detection ability, and uniformly handle multi-scale defects through multi-level feature integration [24–25]. However, it requires step-by-step optimization of feature extraction, weight fusion, and reconstruction modules, which can easily lead to local optima, and the autoencoder tends to learn the “average normal mode”, which may incorrectly repair defect areas and result in insignificant reconstruction errors [26]. The discriminator of GANs can force the generator to output more realistic normal samples, and the differences in defect areas are more significant because they cannot be perfectly reconstructed. Moreover, it can generate realistic defect samples, expand the diversity of training data, enhance the model’s generalization ability to unknown defects, and effectively improve the generalization and detection efficiency of IDD methods [27–28]. Therefore, this study introduces GANs for UIIDD. Fig 4 shows the training process of GANs.

In the Fig 4, the core training mechanism of GANs is to synthesize images through latent space vectors and noise through a generator, and input them together with real data into a discriminator for authenticity discrimination. The discrimination results are optimized through “fine-tuning training” feedback to force the generated images to approximate the true distribution. The global optimization objective function for GANs training is shown in equation (8).


$${min}_{G}\;{max}_{D}V\left(D,G\right)={𝔼}_{x\;p_{\text{data}}\left(x\right)}\left[logD\left(x\right)\right]+{𝔼}_{z\;p_{z}\left(z\right)}\left[log\left(1-D\left(G\left(z\right)\right)\right)\right]$$ (8)

In equation (8), ${min}_{G}$ is the minimization operation of generator G, ${max}_{D}$ is the maximization operation of discriminator D, and V(D,G) is the objective function. 𝔼 is the expected operator, representing the probability weighted average of a random variable. $x\;p_{\text{data}}\left(x\right)$ is x, which is a sample sampled from the real data distribution $p_{\text{data}}\left(x\right)$. $z\;p_{z}\left(z\right)$ is z, which is a sample sampled from the prior noise distribution $p_{z}\left(z\right)$. D(x) is the output probability of the discriminator for the input sample x. G(z) is the generator output to the noise z, which is the generated data sample. During GANs training, when the discriminator is strong, the loss gradient of the generator tends to approach 0, making it difficult to update the generator. Therefore, to solve the problem of gradient vanishing and accelerate the convergence of the generator, this study directly maximizes the classification error probability of the discriminator on the generated data by introducing the generator non-saturating loss function, as given by equation (9).


$${\hat{L}}_{G}=-{𝔼}_{z\;p_{z}\left(z\right)}\left[log\left(1-D\left(G\left(z\right)\right)\right)\right]$$ (9)

In equation (9), ${\hat{L}}_{G}$ represents the non-saturating loss of the generator, $-{𝔼}_{z\;p_{z}\left(z\right)}$ means equating the minimization loss of the generator to the term in the maximum expectation. However, the adversarial game of traditional GANs can easily lead to gradient vanishing or pattern collapse, and the local receptive field of traditional convolutional layers is difficult to capture long-range dependencies. By dynamically calculating the correlation weights at any position in the feature map, self-AM breaks through the local limitations of convolution operations and can directly associate distant pixels through self-attention layers [29–30]. Therefore, this study introduces self-AM to improve GANs, where scaled dot product attention calculation is the core computing unit of self-attention. It measures the correlation weight between each element and other elements through dot product similarity, and then aggregates information through weight weighting, as given by equation (10).


$$\text{Attention}\left(Q,K,V\right)=\text{softmax}\left(\frac{QK^{T}}{\sqrt{d_{k}}}\right)V$$ (10)

In equation (10), Attention(·) is the self-attention function. Q, K, and V are the query, key, and value matrix. $QK^{T}$ is the dot product similarity matrix, $\sqrt{d_{k}}$ is the scaling factor, and softmax(·) is the row normalization function. This study aims to solve the problem of single attention heads being unable to model complex dependencies in AM and improve the learning ability of position, syntax, and semantic features. Multiple independent scaled dot product attention heads are combined into a multi-head mechanism MultiHead(·), as shown in equation (11).


$$\left\{ \begin{array}{c} @l@\text{MultiHead}\left(Q,K,V\right)=\text{Concat}({\text{head}}_{\text{1}},\ldots ,{\text{head}}_{h})W^{\text{0}}\\ {\text{head}}_{i}=\text{Attention}\left(Q\overset{Q}{\underset{i}{W}},K\overset{K}{\underset{i}{W}},V\overset{V}{\underset{i}{W}}\right) \end{array}\right.$$ (11)

**Fig 5:**
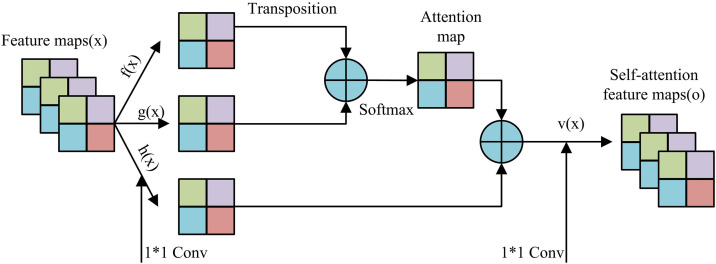
Calculation process of self-attention module.

In equation (11), h is the number of attention heads. ${\text{head}}_{i}$ is the output result of the i -th attention head. $\overset{Q}{\underset{i}{W}}$ and $\overset{K}{\underset{i}{W}}$ are the query and key projection matrix. $\overset{V}{\underset{i}{W}}$ is the value projection matrix. $\text{Concat}({\text{head}}_{\text{1}},\ldots ,{\text{head}}_{h})$ is the concatenation matrix along the feature dimension, and $W^{o}$ is the output projection matrix. In summary, the module calculation process in AM is shown in Fig 5.

In Fig 5, the process of the module first inputs the feature map and generates f(x), g(x), and h(x) through three independent 1 × 1 convolutions. After transpose, f(x) is multiplied by g(x), and then softmax is used to generate an attention feature map. This weight is weighted and fused with h(x), and then convolved by 1 × 1 to output the self-attention feature map. In the process of integrating AM and GANs, to ensure that the data is enhanced and trained stably, this study carries out data preprocessing and model initialization on industrial image data, as shown in equation (12).


$$\left\{ \begin{array}{c} @l@\overset{\left(i\right)}{\underset{\text{aug}}{x}}=\text{T}\left(x^{\left(i\right)}\right)=x^{\left(i\right)}⊕\varepsilon_{\text{noise}}+\gamma_{\text{affine}}\cdot {\text{R}}_{\text{rotate}}\left(x^{\left(i\right)}\right)\\ \overset{\left(0\right)}{\underset{D}{\vartheta }},\overset{\left(0\right)}{\underset{G}{\vartheta }}\text{ U}\left(-a,a\right),a=\sqrt{\frac{\text{6}}{d_{in}+d_{\text{out}}}}\\ \overset{\left(k\right)}{\underset{\text{attn},D}{W}}\leftarrow {\text{Init}}_{\text{Xavier}}\left(0\right),\overset{\left(k\right)}{\underset{\text{attn},G}{W}}\leftarrow {\text{Init}}_{\text{Xavier}}\left(0\right) \end{array}\right.$$ (12)

In equation (12), $\overset{\left(i\right)}{\underset{\text{aug}}{x}}$ is the enhanced image, T(·) is the data augmentation function, and $x^{\left(i\right)}$ is the original training image. $\varepsilon_{\text{noise}}$ is the Gaussian noise matrix, $\gamma_{\text{affine}}$ is the affine transformation strength coefficient, and ${\text{R}}_{\text{rotate}}(\cdot )$ is the random rotation transformation function. $\overset{\left(0\right)}{\underset{D}{\vartheta }}$ and $\overset{\left(0\right)}{\underset{G}{\vartheta }}$ are the initial parameters of discriminator D and generator G.  U(−a,a) is a uniform distribution initialization, which means that the parameters are uniformly sampled from interval [−a,a]. $d_{in}$ and $d_{\text{out}}$ are the input and output dimensions of the neural network layer. $\overset{\left(k\right)}{\underset{\text{attn},D}{W}}$ and $\overset{\left(k\right)}{\underset{\text{attn},D}{W}}$ are the self-attention weights of the k -th layer of the discriminator and generator. ← represents the assignment operation. ${\text{Init}}_{\text{Xavier}}\left(0\right)$ is Xavier initialization, where 0 represents sampling from a zero mean distribution. To distinguish between real images and generator reconstructed images, focus the discriminator on global structural consistency rather than local pixels, and improve sensitivity to large-area defects, this study conducts adversarial training on the discriminator fused with self-attention, as shown in equation (13).


$$\left\{ \begin{array}{c} @l@L_{D}=\begin{array}{c} -{𝔼}_{X\;p_{\text{data}}}\left[logD_{\text{real}}\left(F_{D}\left(x\right)\right)\right]-{𝔼}_{Z\;p_{z}}\left[log\left(1-D_{\text{fake}}\left(F_{D}\left(G\left(z,x\right)\right)\right)\right)\right]\\ +\lambda_{\text{fm}}\cdot {𝔼}_{x\;p_{\text{data}},z\;p_{z}}\left[\left|\right|\phi_{i}\left(SA_{\text{0}}\left(x\right)\right)-\phi_{i}\left(SA_{\text{0}}\left(G\left(z,x\right)\right)\right)|\overset{\text{2}}{\underset{\text{2}}{|}}\right]+\lambda_{\text{reg}}\cdot \Omega \left(\vartheta_{D}\right) \end{array}\\ {𝐅}_{D}(\cdot )={\text{SA}}_{D}(\cdot )⊕\text{ConvNet}(\cdot )\\ {\text{SA}}_{D}\left(x\right)=\text{LayerNorm}\left(\text{softmax}\left(\frac{\left(xW_{Q}\right)(xW_{K}{)}^{T}}{\sqrt{d_{k}}}\right)\cdot \left(xW_{V}\right)+x\right) \end{array}\right.$$ (13)

In equation (13), $L_{D}$ is the total loss function of the discriminator. G(z,x) is the reconstructed image output by the generator. $D_{\text{real}}(\cdot )$ means the classification output head of the discriminator for the real image. $D_{\text{fake}}\left(\cdot \right)$ is the classification output head of the discriminator for generating images. $F_{D}(\cdot )$ is the discriminator feature extractor. ${\text{SA}}_{D}(\cdot )$ is the self-attention feature transformation of the discriminator, LayerNorm(·) is the layer normalization, and $\lambda_{\text{fm}}$ is the feature matching loss weight. $\phi_{i}\left(\cdot \right)$ is the intermediate feature of the l -th layer of the discriminator, $\lambda_{\text{reg}}$ denotes the regularization coefficient, and $\Omega \left(\vartheta_{D}\right)$ is the penalty term of the discriminator parameters. To enhance the reconstruction ability of the generator for normal modes while suppressing defect generation, this study combines the latest research progress in IDD and conducts adversarial optimization training on the generator through multi-level self-attention guidance, as shown in equation (14).


LG=λrecon·(𝔼x~pdatez~pz[∣∣x−G(z,xaug)∣∣1⊙MattnG]+β·𝔼x~pdatez~pz[∣∣Φ(x)−Φ(G(z,xaug))∣∣F2])+λadv·𝔼x~pdatez~pz[−logσ(D(𝐅D(SAD(L4)(G(z,xaug))))]+λreg·(∑k=1Nattn∣∣Wattn,G(k)∣∣1) (14)

In equation (14), ${\text{L}}_{G}$ is the total loss function of the generator. $\lambda_{\text{recon}}$ is the weight coefficient of the feature level reconstruction loss. $\lambda_{\text{adv}}$ is the weight coefficient for combating losses. $\overset{G}{\underset{\text{attn}}{M}}$ is the attention mask matrix generated by the generator’s self-attention module. $W_{\text{attn},G}$ is the trainable weight matrix of the generator’s self-attention module. Finally, this study combines the reconstruction error with the discriminator score to obtain the final industrial image defect detection score, as shown in equation (15).


$$\left\{ \begin{array}{c} @l@S_{\text{anomaly}}\left(x_{\text{test}}\right)={\|x_{\text{test}}-G\left(z,x_{\text{test}}\right)\|}_{\text{1}}⊙A_{\text{attn}}+\delta \cdot \left(1-\sigma \left(F_{D}\left(SA_{D}\left(x_{\text{test}}\right)\right)\right)\right)\\ A_{\text{attn}}=\text{diag}\left(\text{softmax}\left(\frac{\left(x_{\text{test}}W_{K}\right)(x_{\text{test}}W_{Q}{)}^{T}}{\sqrt{d}}\right)\right) \end{array}\right.$$ (15)

**Fig 6:**
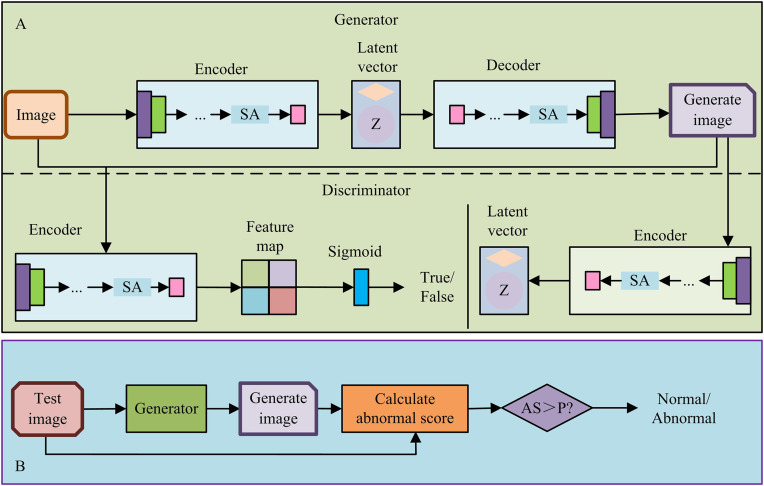
UIIDD model framework integrating self-AM and GANs.

In equation (15), $S_{\text{anomaly}}$ is the image defect score, and $x_{\text{test}}$ is the test image. $A_{\text{attn}}$ is the spatial attention weight matrix. diag is the diagonal of the attention matrix extracted. δ is the weighting coefficient of the discriminator term. In summary, the UIIDD model framework that integrates AM and GANs is shown in Fig 6.

**Fig 7:**
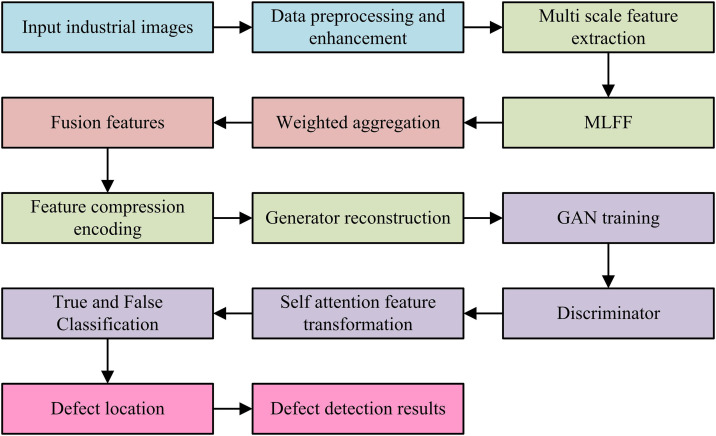
UIIDD process based on autoencoder and GANs.

In Fig 6, the UIIDD model is segmented into a training and a testing phase. During the training phase, the generator learns the normal sample distribution through an encoder-decoder structure with a self-attention module and outputs a reconstructed image. The discriminator synchronously utilizes an encoder enhanced by self-AM, and combines latent vectors with generated images for authenticity discrimination, driving adversarial optimization. During the testing phase, the image to be tested is reconstructed by the generator, and its deviation from the normal mode is quantified through pixel-level anomaly score calculation. The defect classification result is output based on threshold judgment. The process of the unsupervised IDD method based on autoencoder and GANs is shown in Fig 7.

In Fig 7, UIIDD based on autoencoder and GANs takes industrial image input as the starting point, and after data preprocessing and multi-scale feature extraction, enhances the model expression ability through MLFF. Subsequently, an autoencoder is used to compress features into a latent space, and the generator reconstructs normal samples based on this. It introduces an adversarial training mechanism and a discriminator joint self-attention module, dynamically optimizes the generation quality through true false classification, and forces the reconstructed image to approach the normal distribution. During testing, the model generates abnormal heat maps by calculating the local discrepancies between the input and reconstructed images, ultimately achieving precise defect localization.

## 4. UIIDD method verification based on autoencoder and GANs

To systematically verify the performance of the proposed method, this study adopts a unified evaluation process, including data preprocessing, model training, post-processing strategies, and threshold setting. The specific solution is a unified data segmentation, resampling, and post-processing strategy. (1) Data segmentation: Stratified sampling is used to divide the MVTec AD and DAGM datasets into training sets, verification sets, and test sets in a ratio of 7:2:1 to ensure that various defects are distributed consistently in the subsets. (2) Resampling: To address the problem of the scarcity of defect samples, minority class defects are randomly oversampled during the training phase to avoid model bias. (3) Post-processing: Gaussian filtering is performed on the anomaly score map output by the model to suppress noise, and Connected Component Analysis is used to merge adjacent anomaly areas to improve the continuity of defect location. Threshold setting basis (the defect determination threshold is comprehensively determined based on three strategies) (1) Validation set rules: The F1-Score threshold is maximized by grid search (step size 0.01) on the validation set. (2) Youden’s Index: The threshold is maximized corresponding to sensitivity + specificity – 1. (3) Fixed operating point: On the premise that the recall rate is not less than 90%, the threshold with the highest precision rate is selected as the fixed operating point, which is suitable for real-time detection scenarios.

### 4.1 Performance testing of UIIDD method

To verify the performance of the unsupervised IDD method based on autoencoders and GANs, a simulation experiment platform WAs constructed. The hardware environment used NVIDIA Tesla V100 and A100 GPUs, equipped with 32GB/40GB video memory. The system CPU memory was 256GB DDR4. The software environment was based on Docker container deployment, using CUDA 11.4 and cuDNN 8.2.4 to accelerate calculations. The deep learning framework was PyTorch 1.12.1 and TensorFlow 2.10.0, and image processing relied on OpenCV 4.5.5. Its specific configuration is shown in Table 1.

**Table 1:** Test environment and specific configuration.

Testing environment	Specific configuration
GPU	NVIDIA Tesla V100 32GB/ A100 40GB
GPU driver version	470.82.01
CUDA version	11.4
cuDNN version	8.2.4
CPU	Intel Xeon Gold 6248R
Memory	256GB DDR4
Storage	NVMe SSD 2TB
Deep learning framework	PyTorch 1.12.1/ TensorFlow 2.10.0
Image processing	OpenCV 4.5.5
Environmental management	Docker 20.10.7

**Fig 8:**
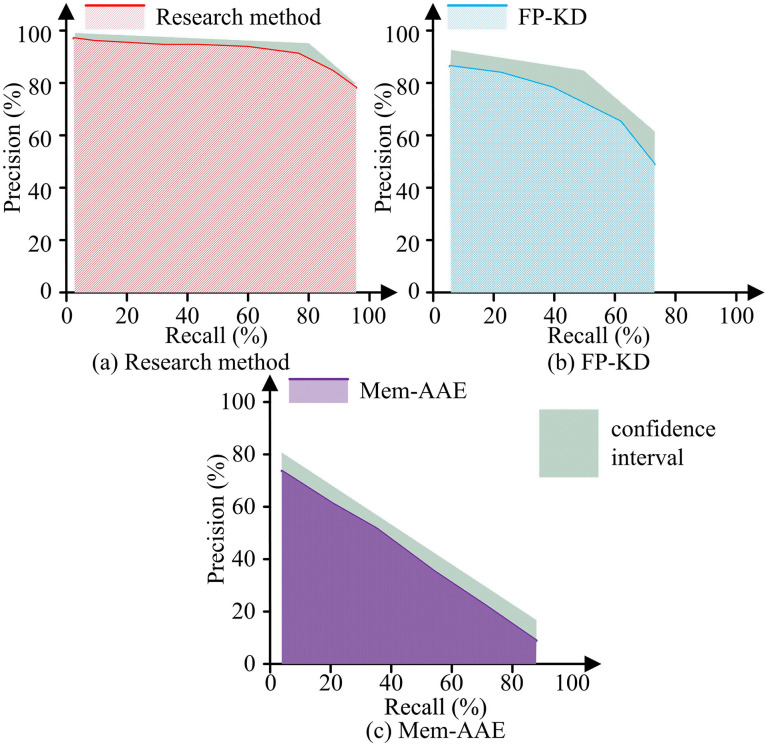
RP curves of different methods.

In Table 1, the specific configurations in the table were used for performance testing. To evaluate the stability and reproducibility of the proposed method, all experiments were independently repeated three times, using different random seeds each time. The study used the MVTec AD dataset for testing. The research methods were compared with the Feature Pyramid Knowledge Distillation (FP-KD) framework and the Memory-Enhanced Attention Autoencoder (Mem-AAE) framework. The comparison of Recall-Precision (RP) curves and Area Under the Curve (AUC) for the three methods is shown in Fig 8.

**Fig 9:**
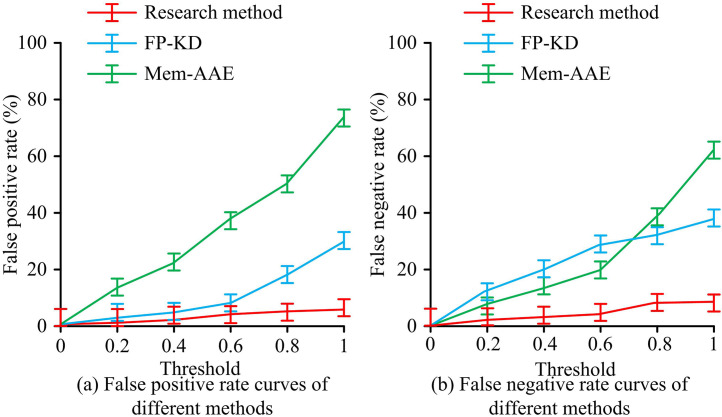
FPR and FNR at different thresholds.

In Fig 8(a), the RP curve of the research method was biased towards the upper left corner. When the recall rate was 95.6%, the precision of the research method was as high as 80.3%, and the AUC of its RP was close to a rectangle with an area of about 93.6 ± 0.5%. In Fig 8(b), the RP curve of FP-KD showed a slow downward trend. When the recall rate was 72.4%, the precision of FP-KD was 50.3%, and its AUC was close to a quarter circle, with a value of about 63.9 ± 1.2%. In Fig 8(c), the RP curve of Mem-AAE rapidly decreased with a slope close to 1, and the AUC of Mem-AAE’s RP was close to a triangle, with a value of approximately 42.6 ± 0.9%. Overall, the research method had better defect capture capability and detection precision. The changes in False Positive Rate (FPR) and False Negative Rate (FNR) of the three methods with increasing threshold are compared, as exhibited in Fig 9.

**Fig 10:**
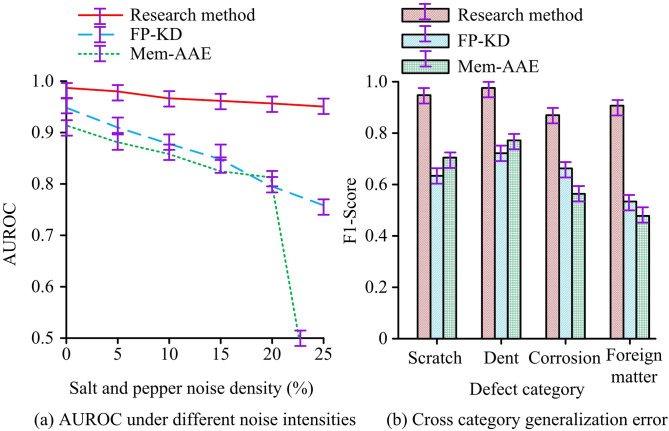
AUROC values and cross category generalization errors under different noise intensities.

In Fig 9, the FPR and FNR of the three methods increased with the increase in the threshold. In Fig 9(a), the FPR of the research method showed the smallest increase, with only a 5.2 ± 0.1% increase when the threshold was increased to 1. When the threshold was increased to 1, the FPRs of FP-KD and Mem-AAE were 32.3 ± 0.2% and 78.9 ± 0.2%. In Fig 9(b), as the threshold increased, the increase in FNR of the research method was relatively small, only 6.4 ± 0.2% when the threshold increased to 1. The increase in FP-KD was significant when the threshold was less than 0.6, and when the threshold was 0.6, its FNR reached as high as 28.4 ± 0.1%. The FNR of Mem-AAE increased significantly when the threshold was greater than 0.6, and when the threshold was 1, its FNR reached as high as 64.5 ± 0.3%. Compared to comparative methods, research methods had stronger defect sensitivity and generation discrimination synergy. The comparison of Area Under ROC Curve (AUROC) values and F1-Score values for detecting different types of defects using three methods under different salt and pepper noise densities is shown in Fig 10.

In Fig 10(a), the AUROC of the three methods decreased with increasing Salt and Pepper Noise Density (S&PND). The decrease in the research method was minimal, with an AUROC value of 0.970 ± 0.004 when the S&PND increased to 25%. When the S&PND increased to 25%, the AUROC value of FP-KD decreased to 0.750 ± 0.002. The AUROC value of Mem-AAE showed an abnormal decrease when the S&PND was 20%. In Fig 10(b), the F1-Score values of the research method were 0.980 ± 0.005, 0.990 ± 0.001, 0.890 ± 0.003, and 0.91 ± 0.001 for defect types such as scratches, dents, corrosion, and foreign objects. The other two methods showed significantly lower F1 Score values in detecting different types compared to the research method. Overall, the research method had stronger robustness and generalization. The proposed UIIDD method based on autoencoder and GANs had good defect capture ability, detection precision, generation discrimination synergy, robustness, and generalization.

### 4.2 Ablation study

To validate the individual and joint contributions of the proposed modules, we conduct an ablation study comparing the following five model variants: Model A (Baseline GAN): A standard GAN with a conventional encoder-decoder as the generator and a CNN-based discriminator. Model B (MDAAE): The proposed multi-level deep feature adaptive fusion autoencoder. Model C (self-attention-enhanced GAN (SA-GAN)): A GAN enhanced with a self-attention mechanism in the discriminator. Model D (MDAAE + Baseline GAN): The proposed MDAAE generator combined with a Baseline GAN-based discriminator. This variant isolates the contribution of the advanced generator from the enhanced discriminator. Model E (MDAAE + SA-GAN): The complete framework integrating both MDAAE and the SA-GAN. All models are evaluated on the MVTec AD dataset under identical experimental settings. Key metrics include AUROC, F1-Score (scratch category), inference time, GPU memory usage, and FID stability. The results are summarized in Table 2.

**Table 2:** Ablation study results on MVTec AD dataset.

Model	AUROC (%)	F1-Score	Inference Time (ms)	GPU Memory (GB)	FID
A: Baseline GAN	85.2 ± 0.36	0.840 ± 0.006	195 ± 8	2.8 ± 0.2	5.2 ± 0.3
B: MDAAE	89.7 ± 0.4	0.910 ± 0.004	210 ± 6	3.1 ± 0.2	4.5 ± 0.2
C: SA-GAN	88.3 ± 0.5	0.890 ± 0.005	205 ± 7	2.9 ± 0.2	4.8 ± 0.3
D: MDAAE + Baseline GAN	91.5 ± 0.4	0.940 ± 0.004	208 ± 6	3.1 ± 0.2	3.8 ± 0.2
E: MDAAE + SA-GAN	93.6 ± 0.5	0.980 ± 0.005	201 ± 5	3.0 ± 0.2	3.0 ± 0.3

In Table 2, Model B shows significant improvement over baseline in multi-scale defect capture, attributed to the hierarchical feature fusion in MDAAE. Model C excels in detecting structural anomalies due to the self-attention mechanism’s ability to model long-range dependencies. Model D, which pairs MDAAE with a Baseline discriminator, achieves notable gains over Models B and C, underscoring the strength of the MDAAE generator. However, Model E (MDAAE + SA-GAN) outperforms all variants, demonstrating that the combination of MDAAE and SA-GAN discriminator is complementary and jointly essential for achieving the highest accuracy in unsupervised defect detection.

### 4.3 Actual application effect of UIIDD method

**Fig 11:**
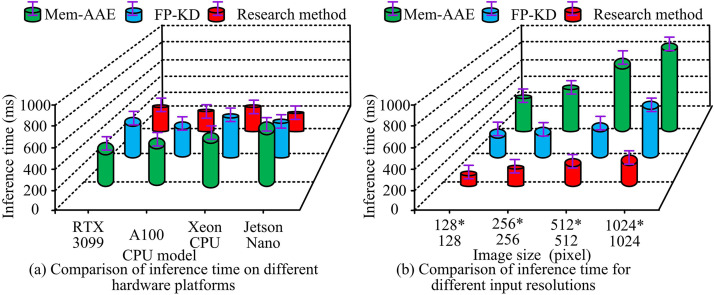
Reasoning time under different hardware platforms and input resolutions.

Based on validating the performance of UIIDD method based on autoencoder and GANs, this study further validated the practical application effect. This study used the DAGM texture dataset to build the “AIGAN ADBench” IDD validation platform. To further verify the applicability of the model in actual industrial environments, this study deployed a detection system in the semiconductor packaging production line of the cooperative enterprise, and conducted field tests on typical defects such as micro scratches on the wafer surface and solder joint virtual soldering. The research methods were compared with FP-KD and Mem-AAE. The comparison of inference time of three methods on different hardware platforms and input image resolutions is shown in Fig 11.

**Fig 12:**
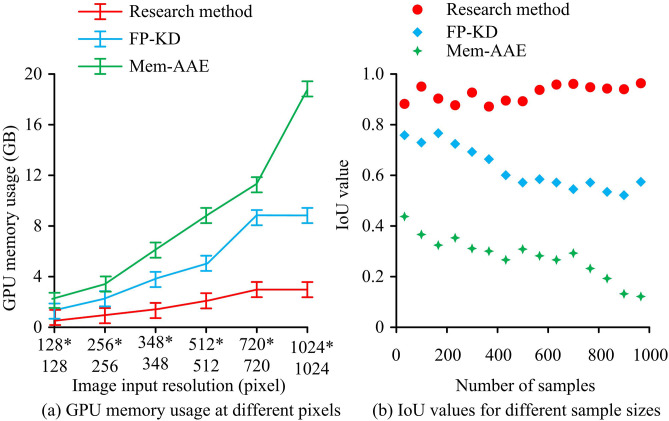
GPU memory usage and IoU value distribution with different pixel sizes.

In Fig 11, the three methods had different inference times for different Central Processing Unit (CPU) models and image sizes. In Fig 11(a), when the CPU models were PTX 3099, A100, Xeon CPU, and Jetson Nano, the inference time of the research method was 150 ± 4 ms, 95 ± 2 ms, 200 ± 3 ms, and 87 ± 5 ms. The inference time was greatly longer than the research method in various CPU models. In Fig 11(b), the inference time of the three methods increased with the increase of image size, while the research method showed the slowest increasing trend. The inference time of the research method was 55 ± 1 ms and 201 ± 5 ms for image sizes of 128*128 pixels and 1024*1024 pixels. Other methods had significantly larger inference times for each size, and the increasing trend was also greater. Overall, the research method had better deployment adaptability and real-time processing capability. Three methods were compared in terms of memory usage in Graphics Processing Units (GPUs) with different image input resolutions, as well as the distribution of Intersection over Union (IoU) values at different sample sizes, as shown in Fig 12.

**Fig 13:**
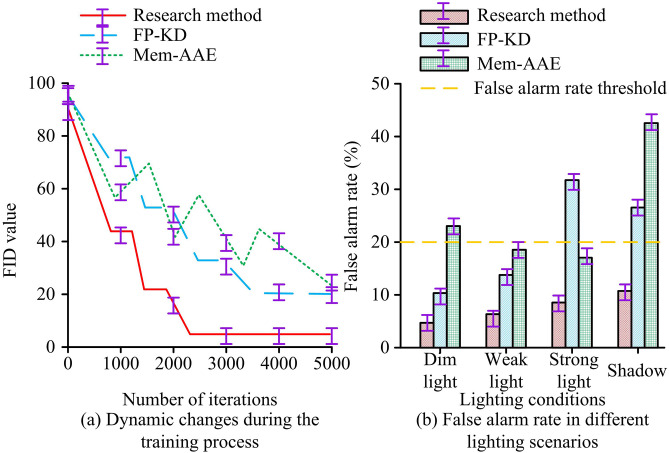
FAR of dynamic changes during the training process and different lighting scenarios.

In Fig 12(a), the GPU memory usage of the three methods gradually increased with the increase of image input resolution. The increase in research methods was minimal, with a GPU memory usage of 1.0 ± 0.1 GB when the input image resolution was 128*128 pixels and a stable value of 3.0 ± 0.2 GB when the resolution was 720*720 pixels. The GPU memory usage of FP-KD reached a stable value of 8.0 ± 0.1 GB when the input image resolution was 720*720 pixels, while Mem-AE increased to 19.0 ± 0.2 GB when the resolution was 1024*1024 pixels. In Fig 12(b), the IoU value distribution of the research method was generally concentrated between 0.85–1.0 for different sample sizes. As the sample size increased, its IoU value fluctuated less (Standard deviation<0.05). The fluctuation amplitude of the IoU value distribution of FP-KD with increasing sample size was 0.26 ± 0.03. The IoU value distribution of Mem-AAE was generally below 0.44. The research method had stronger resource consumption scalability and defect pixel-level localization accuracy. The FID values and False Alarm Rates (FARs) of the three methods at different iteration times and under different lighting intensities are shown in Fig 13.

**Fig 14:**
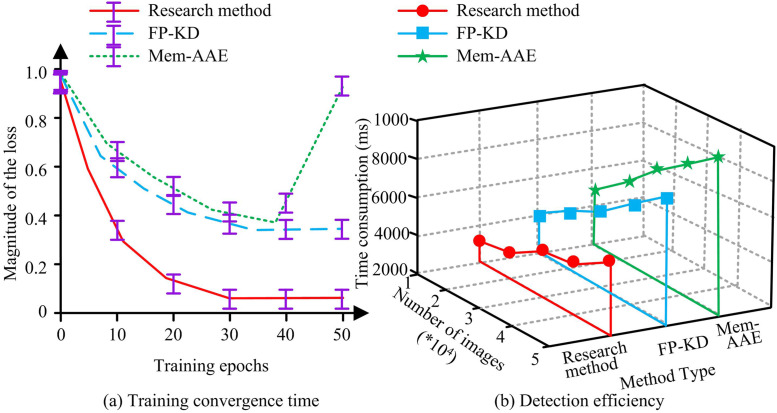
Convergence time and detection efficiency during training.

In Fig 13(a), the convergence speed of FID values for the three methods varied with the number of iterations. The FID value of the research method converged rapidly in the first 2,000 iterations and fully converged by 2,230 iterations, with a stable FID value of 3.0 ± 0.2. The FID value of FP-KD only fully converged after 3,610 iterations. The FID value of Mem-AE fluctuated continuously with increasing iterations and could not converge. In Fig 13(b), the FAR threshold of the system under different lighting conditions was 20%. The FAR of the research method was below the threshold under different lighting conditions. The FAR of the research method was 4.7 ± 0.6% under low light conditions, 7.8 ± 0.7% under low light conditions, 8.6 ± 0.7% under strong light conditions, and 11.5 ± 0.5% under shadow conditions. The FAR of the comparison method was significantly larger under different lighting conditions. The research method had better reliability and illumination robustness. Fig 14 shows the convergence of the loss values of three methods with training epochs and the detection time for different numbers of images.

In Fig 14(a), the detection loss values of the three methods varied with the convergence velocity of the training epochs. The convergence speed of the research method was the fastest, and the detection loss value fully converged at the 30th training, with a stable value of 0.080 ± 0.005. The detection loss value of FP-KD only fully converged at the 33rd training session, while Mem-AE showed an abnormal increase at the 37th training session. In Fig 14(b), the detection time of the three methods increased with the growth of the number of images. The trend of increasing research methods was the smallest. When the number of images was 10,000 and 50,000, the detection time of the research method was 3,100 ± 10 ms and 5,200 ± 20 ms, with the latter only increasing by 2,100 ± 10 ms. When the number of images was 50,000, the detection time of FP-KD was 6,800 ± 30 ms. When increasing from 10,000–50,000 sheets, the detection time of Mem-AAE increased by 3,000 ± 20 ms. Overall, compared with the comparative method, the research method had better reconstruction ability and detection efficiency. Finally, the study selected typical defect samples (scratch defect samples, dent defect samples, and corrosion defect samples) from the MVTec AD dataset for visual analysis. The results showed that the reconstructed image was highly consistent with the original image in the normal area, while obvious reconstruction errors occurred in the defect area. The abnormal heat map could accurately highlight the defect area and was highly consistent with the actual defect location. Under strong light and shadow conditions, the heat map could still locate defects stably, verifying the illumination robustness. Taken together, the UIIDD method based on autoencoders and GAN had good defect location accuracy, reliability, illumination robustness, reconstruction ability, and detection efficiency.

### 4.4 Comparison with various unsupervised AD benchmark models

To further verify the comprehensive performance of the proposed method, this study selected the current mainstream UAD benchmark models for comparison, including PaDiM, SPADE, PatchCore, CFLOW-AD, DRAEM, CutPaste, FastFlow, and RD4AD. All benchmark models were run under the same experimental setup with 95% confidence intervals, and performance was evaluated using the MVTec AD dataset. The results are shown in Table 3.

**Table 3:** Research methods and benchmark model performance comparison.

Model	AUROC (%)	F1-Score (Scratch)	Inference Time (ms)	GPU Video memory (GB)	FID stability value	FAR of strong light (%)	Reference
The proposed method	93.6 ± 0.5	0.980 ± 0.005	201 ± 5	3.0 ± 0.2	3.0 ± 0.3	8.6 ± 0.7	This study
PaDiM	89.2 ± 0.6	0.910 ± 0.003	245 ± 9	4.2 ± 0.3	4.5 ± 0.6	12.3 ± 0.8	[31]
SPADE	88.5 ± 0.5	0.890 ± 0.008	278 ± 6	5.1 ± 0.6	5.2 ± 0.1	14.1 ± 0.2	[32]
PatchCore	90.1 ± 0.4	0.920 ± 0.009	220 ± 8	6.8 ± 0.4	4.0 ± 0.3	10.8 ± 0.9	[33]
CFLOW-AD	87.8 ± 0.2	0.870 ± 0.010	265 ± 5	3.5 ± 0.2	5.8 ± 0.2	15.2 ± 0.5	[34]
DRAEM	86.5 ± 0.3	0.850 ± 0.005	310 ± 12	7.2 ± 0.3	6.5 ± 0.5	16.7 ± 0.6	[35]
CutPaste	84.9 ± 0.1	0.830 ± 0.002	290 ± 10	4.0 ± 0.1	7.1 ± 0.8	18.9 ± 0.2	[36]

In Table 3, the research method outperformed all comparative baseline models in key indicators such as AUROC, F1-Score, inference efficiency, video memory occupancy, and FID convergence. The FAR under bright light conditions was significantly lower than that of other baseline models, showing stronger light robustness. In addition, while maintaining high accuracy, the inference time and GPU video memory usage of the research method were significantly lower than those of other baseline models, indicating that it has better adaptability to industrial deployment.

## 5. Discussion

### 5.1 Significance of research results

The proposed UIIDD framework, which integrated MDAAE and SA GANs, solved the scale sensitivity problem of traditional methods on complex texture defects by transforming defect detection into a “normal mode reconstruction bias analysis” problem, achieving technological paradigm innovation. The model maintained an inference speed of 87 ± 5 ms on edge devices, with a stable memory usage of 3.0 ± 0.2 GB, solving the problem of memory explosion in high-resolution images using FP-KD and other methods. It provided technical support for real-time detection of production lines and enhanced industrial implementation value. By eliminating reliance on defective samples, social and economic benefits have been increased.

### 5.2 Comparison with existing research

Compared to the single-scale feature pyramid of FP-KD, the multi-level fusion mechanism of MDAAE achieved a cross-category defect F1-Score mean of 0.94, which was 51.8% higher than Mem-AAE and achieved a breakthrough in detection performance [7]. When the S&PND was 25%, the AUROC of the research method still remained at 0.970 ± 0.004, significantly higher than the FP-KD method, which compensated for the “noise sensitivity” defect pointed out by Maggipinto M et al. [9]. Expanding the biased knowledge framework to the unsupervised domain and achieving “defect region focusing” through self-attention weights solved the problem of “generator ignoring local anomalies” [30].

### 5.3 Limitations of the research

The research method showed that the FAR increased to 8.6 ± 0.7% under strong light conditions and 11.5 ± 0.5% under shadow conditions. However, due to the model’s dependence on pixel-level reconstruction errors, uneven lighting in industrial sites can cause grayscale differences in normal textures, leading to misjudgment as defects. Moreover, this study relies on reconstruction bias detection for defect types not seen in the training data. However, if the defect morphology is similar to normal texture, the anomaly score may be lower than the threshold. Future work can enhance the stability of structural information to lighting changes through the fusion of multimodal perception and physical modeling, and achieve rapid adaptation through open set learning and uncertainty quantification, utilizing a small number of new defect samples on the production line.

### 5.4 Considerations for lighting robustness design

Industrial environments often exhibit complex illumination conditions, including global brightness shifts, shadows, and specular reflections, which can cause grayscale distribution deviations and interfere with defect detection. This study adopts a preprocessing-based image filtering and normalization strategy (e.g., local contrast normalization, homomorphic filtering) for the following reasons: (1) Feasibility under Unsupervised Setting: Representation-level methods (e.g., illumination-invariant features or domain adaptation) typically require labeled data or complex adversarial training, which are difficult to stabilize in unsupervised, low-sample scenarios. (2) Computational Efficiency and Deployment Simplicity: Preprocessing filters introduce minimal overhead during inference, are easy to integrate into existing detection pipelines, and are suitable for real-time industrial applications. (3) Targeted Handling of Known Disturbances: By enhancing local contrast and homogenizing illumination, preprocessing can effectively suppress grayscale gradients caused by lighting, thereby highlighting the local structural differences of real defects. (4) Synergy with Subsequent Modules: Preprocessed images facilitate normal pattern reconstruction by the Autoencoder and GAN, reducing reconstruction errors due to illumination variations that could be mistaken as anomalies. Compared to end-to-end representation learning schemes, although our strategy does not theoretically eliminate all illumination effects, it demonstrates stable performance on datasets such as MVTec AD and DAGM (e.g., FAR of only 8.6% under strong light), achieving a favorable balance between efficiency and effectiveness.

To further justify the necessity and effectiveness of the proposed preprocessing-based illumination robustness strategy, we compare it with two alternative representation-level or data-centric approaches under the unsupervised setting. Contrastive Learning (Weakly Supervised Scenario): Contrastive learning methods typically rely on constructing positive and negative pairs to learn illumination-invariant representations. However, in unsupervised industrial defect detection, obtaining reliable negative samples (e.g., defect-free images under varying illumination) is challenging. Moreover, contrastive learning introduces additional training complexity and computational overhead, which may hinder real-time deployment on edge devices. Data-Centric Unsupervised Approach: An alternative is to include illumination-augmented normal samples (e.g., low-light or shadowed images) in the training set, allowing the autoencoder to reconstruct them as part of the normal distribution. While this may reduce reconstruction errors caused by lighting variations, it is difficult to cover all possible lighting conditions in practice. Furthermore, over-augmentation may blur the boundary between normal variations and actual defects, reducing defect sensitivity.

In contrast, our method adopts a lightweight preprocessing pipeline consisting of local contrast normalization and homomorphic filtering. This approach requires no additional labels, maintains unsupervised integrity, and introduces minimal inference overhead. The results show that this method achieves a low false positive rate under both strong light (8.6%) and shadow conditions, maintaining defect localization accuracy while demonstrating excellent lighting robustness.

## 6. Conclusion

In response to the problems of insufficient detection ability and poor generalization of traditional detection methods in small sample scenarios, this study innovatively proposed a UIIDD method based on autoencoder and GANs. This study achieved precise localization of industrial image defects by constructing MDAAE and introducing self-AM improved GANs. In the experiment, when the threshold was increased to 1, the FPR of the research method only increased by 5.2 ± 0.1%. When the S&PND increased to 25%, the AUROC of the research method was 0.970 ± 0.004. In practical application, the inference time of the research method was 55 ± 1 ms and 201 ± 5 ms for image sizes of 128*128 pixels and 1024*1024 pixels. The distribution of IoU values for different sample sizes was generally concentrated between 0.85–1.0. At the 30th training session, the detection loss value of the research method fully converged, with a stable value of 0.080 ± 0.005. Overall, the research method has lifted the positioning accuracy, reliability, illumination robustness, and detection efficiency of UIIDD.

## Supporting information

S1 File Minimal data set definition. (DOC)

## Abbreviations

GANs: Generative Adversarial Networks
UIIDD: Unsupervised Industrial Image Defect Detection
MDAAE: Multi-level Deep feature Adaptive fusion AutoEncoder
PTCNBN: Pre-Trained Convolutional Neural Backbone Networks
AM: Attention Mechanism
AUC: Area Under the Curve
FID: Fréchet Inception Distance
IDD: Industrial Defect Detection
GAP: Global Average Pooling
MLDF: Multi-level Deep Feature
UAD: Unsupervised Anomaly Detection
FP-KD: Feature Pyramid Knowledge Distillation
Mem-AAE: Memory-Enhanced Attention Autoencoder
FPR: False Positive Rate
FNR: False Negative Rate
AUROC: Area Under ROC Curve
S&PND: Salt and Pepper Noise Density
CPU: Central Processing Unit
IoU: Intersection over Union
