# Supplementary material for: Unsupervised industrial image defect detection based on autoencoder and GANs
Source: PLoS One. 2026 Apr 10;21(4):e0346637. doi: 10.1371/journal.pone.0346637 (PMC13068286; doi:10.1371/journal.pone.0346637)
Supplement: S1 File — (DOC) [file pone.0346637.s001.doc]

**The data in Figure** 8

| Recall (%) | Precision (%) | | |
| --- | --- | --- | --- |
|  | Research method | FP-KD | Mem-AAE |
| 0 | - | - | - |
| 20 | 98.2 | 82.1 | 60.2 |
| 40 | 98.1 | 72.2 | 46.3 |
| 60 | 98.0 | 63.4 | 29.8 |
| 80 | 85.2 | - | 17.9 |
| 100 | - | - | - |

**The data in Figure** 9

| Threshold | False positive rate (%) | | | False negative rate (%) | | |
| --- | --- | --- | --- | --- | --- | --- |
|  | Research method | FP-KD | Mem-AAE | Research method | FP-KD | Mem-AAE |
| 0 | 0 | 0 | 0 | 0 | 0 | 0 |
| 0.2 | 0.6 | 2.5 | 15.1 | 1.2 | 15.3 | 8.2 |
| 0.4 | 2.4 | 3.6 | 23.2 | 1.6 | 20.1 | 13.6 |
| 0.6 | 3.2. | 10.2 | 38.6 | 1.5 | 28.4 | 19.8 |
| 0.8 | 3.9 | 18.4 | 50.3 | 5.8 | 32.2 | 40.2 |
| 1.0 | 5.5 | 32.3 | 78.9 | 6.4 | 38.4 | 64.5 |

**The data in Figure** 10

| Salt and pepper noise density (%) | AUROC | | |
| --- | --- | --- | --- |
|  | Research method | FP-KD | Mem-AAE |
| 0 | 0.992 | 0.951 | 0.921 |
| 5 | 0.985 | 0.902 | 0.875 |
| 10 | 0.983 | 0.863 | 0.852 |
| 15 | 0.979 | 0.846 | 0.843 |
| 20 | 0.973 | 0.821 | 0.822 |
| 25 | 0.970 | 0.750 | - |
| Defect category | F1-Score | | |
|  | Research method | FP-KD | Mem-AAE |
| Scratch | 0.98 | 0.62 | 0.70 |
| Dent | 0.99 | 0.72 | 0.77 |
| Corrosion | 0.89 | 0.66 | 0.56 |
| Foreign matter | 0.91 | 0.54 | 0.47 |

**The data in Figure** 11

| CPU model | Inference time (ms) | | |
| --- | --- | --- | --- |
|  | Research method | FP-KD | Mem-AAE |
| RTX 3099 | 150 | 232 | 352 |
| A100 | 95 | 206 | 413 |
| Xeon CPU | 200 | 315 | 569 |
| Jetson Nano | 87 | 298 | 645 |
| Image size | Inference time (ms) | | |
|  | Research method | FP-KD | Mem-AAE |
| 128*128 | 55 | 192 | 323 |
| 256*256 | 72 | 231 | 489 |
| 512*512 | 186 | 289 | 612 |
| 1024*1024 | 201 | 445 | 795 |

**The data in Figure 12**

| Image input resolution (Pixel) | GPU memory usage (GB) | | |
| --- | --- | --- | --- |
|  | Research method | FP-KD | Mem-AAE |
| 128*128 | 1.0 | 1.5 | 2.5 |
| 256*256 | 1.3 | 2.5 | 3.5 |
| 348*348 | 2.1 | 3.0 | 6.0 |
| 512*512 | 2.5 | 4.0 | 8.5 |
| 720*720 | 3.0 | 8.0 | 11.5 |
| 1024*1024 | 3.0 | 8.0 | 19.0 |
| Number of samples | IoU value | | |
|  | Research method | FP-KD | Mem-AAE |
| 0 | 0.90 | 0.76 | 0.45 |
| 200 | 0.90 | 0.73 | 0.36 |
| 400 | 0.87 | 1.60 | 0.27 |
| 600 | 0.95 | 0.58 | 0.27 |
| 800 | 0.94 | 0.54 | 0.20 |
| 1000 | 0.97 | 0.58 | 0.13 |

**The data in Figure** 13

| Number of iterations | FID value | | |
| --- | --- | --- | --- |
|  | Research method | FP-KD | Mem-AAE |
| 0 | 90 | 95 | 95 |
| 1000 | 42 | 71 | 60 |
| 2000 | 15 | 52 | 43 |
| 3000 | 3 | 28 | 42 |
| 4000 | 3 | 20 | 37 |
| 5000 | 3 | 20 | 24 |
| Lighting conditions | False alarm rate (%) | | |
|  | Research method | FP-KD | Mem-AAE |
| Dim light | 4.7 | 10.1 | 22.6 |
| Weak light | 7.8 | 12.6 | 18.9 |
| Strong light | 8.6 | 32.1 | 16.3 |
| Shadow | 11.5 | 26.3 | 42.5 |

**The data in Figure** 14

| Training epochs | Magnitude of the loss | | |
| --- | --- | --- | --- |
|  | Research method | FP-KD | Mem-AAE |
| 0 | 0.99 | 0.99 | 0.99 |
| 10 | 0.35 | 0.63 | 0.66 |
| 20 | 0.12 | 0.42 | 0.45 |
| 30 | 0.08 | 0.37 | 0.39 |
| 40 | 0.08 | 0.33 | 0.51 |
| 50 | 0.08 | 0.33 | 0.94 |
| Number of images (*10^4^) | Time consumption (ms) | | |
|  | Research method | FP-KD | Mem-AAE |
| 1 | 3100 | 4080 | 5000 |
| 2 | 3180 | 4650 | 5700 |
| 3 | 3850 | 5230 | 6550 |
| 4 | 4120 | 5960 | 7300 |
| 5 | 5200 | 6800 | 8000 |
